# Bedsided Transcranial Sonographic Monitoring for Expansion and Progression of Subdural Hematoma Compared to Computed Tomography

**DOI:** 10.3389/fneur.2018.00374

**Published:** 2018-05-28

**Authors:** Wolf-Dirk Niesen, Michael Rosenkranz, Cornelius Weiller

**Affiliations:** ^1^Department of Neurology, University Medical Center, University of Freiburg, Freiburg, Germany; ^2^Department of Neurology, Albertinen Hospital Hamburg, Teaching Hospital of the University of Hamburg, Hamburg, Germany

**Keywords:** subdural hematoma, neurotrauma, neurocritical care, transcranial sonography, monitoring

## Abstract

**Introduction:**

Transcranial high-resolution ultrasonography reliably allows diagnosis and monitoring of intracerebral hemorrhage in adults. Sonographic monitoring of subdural hematoma (SDH) has not been evaluated in adults so far. This study investigates the reliability of transcranial gray-scale sonography (TGS) in monitoring acute and chronic SDH in adults.

**Methods:**

TGS was performed in 47 consecutive patients with either acute or chronic SDH confirmed by cerebral CT. Four patients were excluded due to insufficient bone window. After identification of SDH in TGS extent was measured and correlated with extent of SDH on cerebral computer tomography (CCT). If possible measurement was performed at least on 2 days to evaluate the possibility to monitor SDH with TGS.

**Results:**

In 43 patients with SDH, 76 examinations were performed with 2 examinations in 23 patients and 3 examinations in 10 patients. Overall extent of SDH correlated significantly between TGS and CCT (*r* = 0.962). Accordingly correlation was high during each single examination time point. In patients in need for surgical evacuation sonographic measurement yielded a sensitivity of 90.9% and specificity of 93.8% in predicting surgical evacuation (*p* < 0.001).

**Discussion:**

Imaging of SDH with TGS is possible in patients with SDH and extent of SDH correlates significantly between TGS and CCT during initial as well as during follow-up examination. Thus monitoring of SDH with TGS at patients’ bedside is possible.

## Introduction

Subdural hematoma (SDH) is a common complication of major traumatic head injury, as well as in recurrent minor head trauma. In case of minor SDH without a sign of mass effect and without clinical symptoms monitoring of SDH without any surgical intervention is feasible (1). Cerebral computer tomography (CCT) currently is the method of choice to monitor SDH (2). Consecutively especially in critically ill patients with limited clinical monitoring serial CCT is performed leading to patient transportation which may place patients at risk. Thus, there is a need for other monitoring techniques that may be applied at patients’ bedside.

In infants, transcranial high-resolution ultrasonography through the non-ossified fontanel is the method of choice since it reliably allows diagnosis and monitoring of SDH (3, 4). Studies on intracerebral hematomas in adults demonstrated sufficient spatial resolution for diagnosis and measurement of hematoma in transcranial gray-scale sonography (TGS) (5–7) but small cortical hemorrhages could not clearly be distinguished (5). Despite this, SDH may be depicted by TGS (8). Yet, the role of TGS in monitoring SDH and detecting patients in need for surgical evacuation has not been investigated.

This study evaluates the role of sonographic monitoring of acute and chronic SDH by comparing measurement of SDH expansion between TGS and CCT over time, trying to identify patients in need for surgical evacuation accordingly.

## Materials and Methods

47 consecutive patients presenting with acute or chronic SDH were included into the study. Four patients had to be excluded due to insufficient transtemporal bone window.

### Inclusion/Exclusion Criteria

Initial CCT or MRI confirming SDH along the convexity served as the only inclusion criterion. Patients were excluded in other causes of extracerebral space enlargement (e.g., subdural empyema, focal brain atrophy). Also, time interval between TGS and radiologic imaging was not to exceed 12 h and patients were excluded in case of insufficient transtemporal bone window. Finally, patients were not included if patients or patient relatives did not consent to the study.

### Neuroradiological Imaging

Diagnosis and classification regarding acuity of SDH was achieved by either cerebral CT or cerebral MRI following published criteria. Diagnosis and definition of acute or chronic SDH were as follows: on CCT SDH was diagnosed if CCT revealed either a hyperdense (acute SDH), an isodense (hyperacute SDH), or a hypodense homogenous or inhomogeneous (chronic SDH) crescent-shaped fluid collection along the convexity with a sharp margin to the brain parenchyma. In the few cases with MRI-based diagnosis, SDH was defined as typical crescent-shaped fluid collection along the convexity with typical presentation on T1- and T2-weighed images defining the age of SDH.

Extent of SDH on CCT was measured using the same plane that was used for TGS and included lateral extension, e.g., thickness of hematoma only. Measurement was performed by an experienced neuroradiologist. Follow-up CCT examination was performed according to clinical necessity and published clinical procedural criteria.

### Transcranial Gray-Scale Sonography

**Figure 1:**
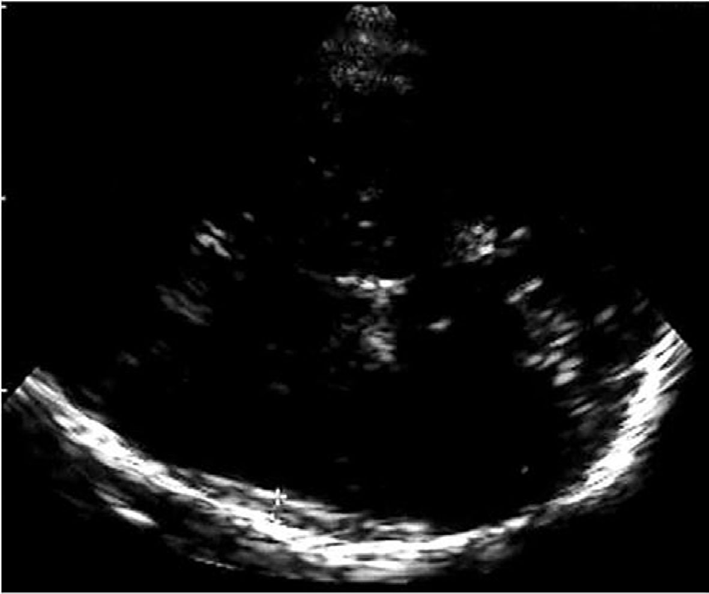
Figure shows the highly echogenic skull opposite to the probe and a highly echogenic membrane clearly distinctable from the skull as sonographic correlate of the dural border of the arachnoid and as a sign of subdural space enlargement.

Sonographic examination was performed using a color-flow ultrasound system feasible for transcranial color-coded duplex sonography (Siemens Sonoline Elegra, Siemens AG, Erlangen; Sonos 5500, Philips, Hamburg) using a 2.0–2.5 MHz phased-array transducer. For insonation, the transtemporal bone window was used in an axial plane, and evaluation of brain parenchyma was performed according to published criteria (9, 10). SDH was visualized with a scanning depth of 16 cm and with progression from the side opposite to the pathology confirmed by CCT. The investigator was notified concerning existence and localization of the SDH but was blinded to extent of SDH. Demonstration of a highly echogenic membrane clearly distinctable from the echogenic opposing skull was defined as the dural border of the arachnoid and as a sign of subdural space enlargement on transcranial gray-scale imaging (Figure 1). Extent of subdural space enlargement was measured by drawing a perpendicular line from the opposing skull to the inside of the echogenic membrane at the broadest part of subdural space enlargement. Sonographic measurement was performed within 12 h of corresponding CCT examinations.

### Statistics

After testing for equal distribution using the Shapiro–Wilk test overall extent of SDH on TGS was correlated with extent on CCT using Pearson’s correlation coefficient. Also, extent of SDH of the single examination was correlated between TGS and CCT using Pearson’s correlation coefficient. Also, the rate of progressing SDH with a need for surgical evacuation was calculated from TGS and sensitivity as well specificity was calculated by ROC analysis. Statistical analysis was performed using SPSS statistical software (SPSS Version 17.0).

### Ethics

This study was carried out in accordance with the recommendations of the ethics committee of Hamburg University and Freiburg University, Germany, with written informed consent from all subjects or their legal guardians. All subjects gave written informed consent in accordance with the Declaration of Helsinki. The protocol was approved by the ethics committee of Freiburg University, Germany.

## Results

47 consecutive patients with SDH confirmed by CCT were included into the study (see Table 1). TGS was performed within 12 h of CCT in all patients. Four patients presented without a transtemporal bone window and were excluded from the study. SDH could be visualized in 43 patients (91.5%). 38 patients presented with acute SDH and 5 with purely chronic SDH.

**Table 1:** Patients’ characteristics.

	*n*	Percentage
All patients	47	8.5
Insufficient transtemporal bone window	4	
Included patients	43	
Examinations overall	76	
1 follow-up	23	53.5
2 follow-ups	10	23.3
Age (mean ± SD)	62.7 ± 16.4 years	
Male	33	76.7
Female	10	23.3
Acuity of subdural hematoma		
Acute	33	76.7
chronic	5	11.6
Acute on chronic	5	11.6
Surgical evacuation	11	25.6

**Figure 2:**
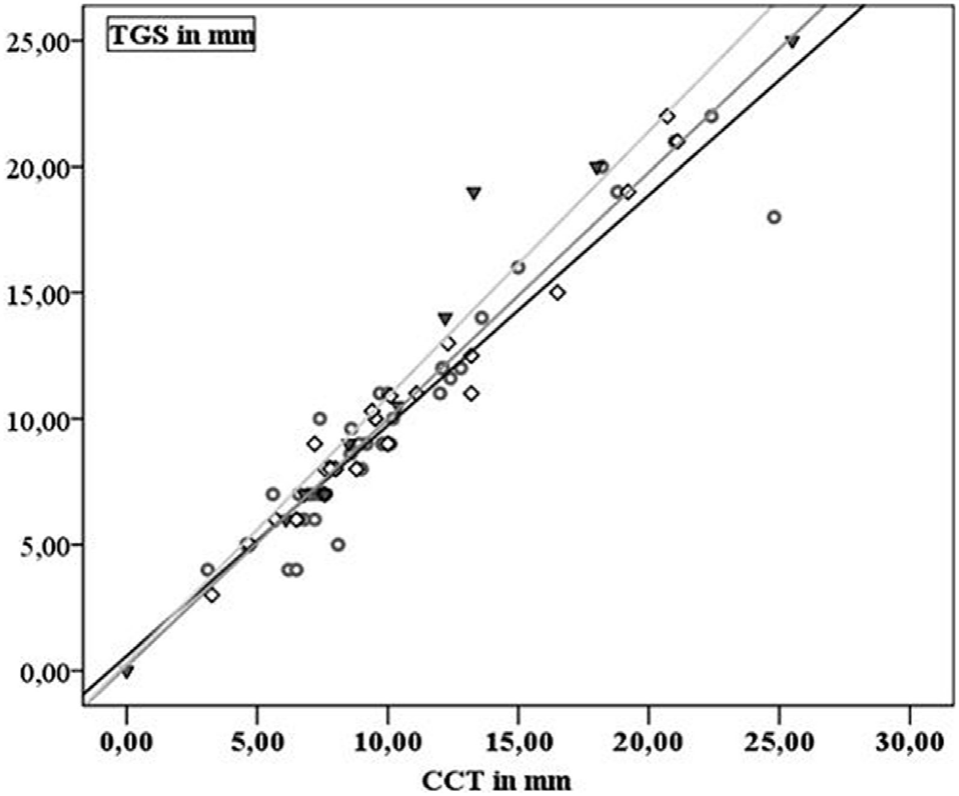
Gray dots and black line: first examination; black squares and dark gray line: second examination; black triangles and gray line: third examination.

**Figure 3:**
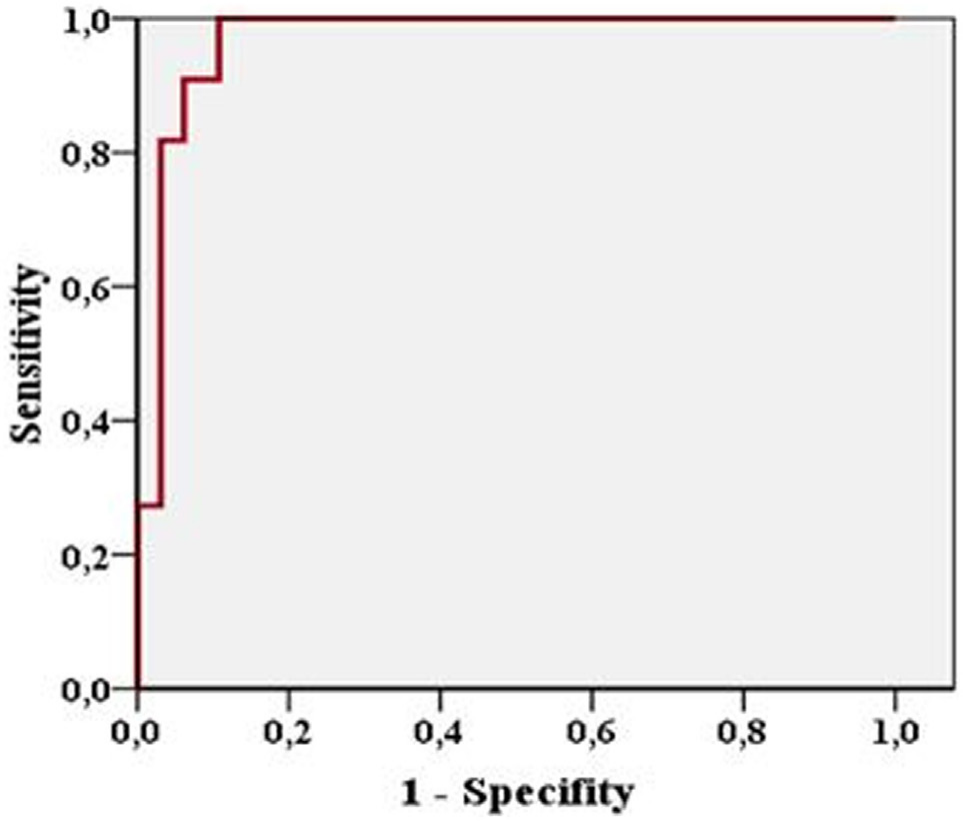
Figure shows high sensitivity and specificity of sonographic measurement in predicting surgical evacuation of subdural hematoma.

Extent of SDH was measured in 43 patients and ranged from 4 to 25 mm on CCT and from 3.1 to 25.5 mm on TGS. In 23 patients, 1 follow-up examination by TGS and CCT was performed and 10 patients received a second follow-up. In 76 examinations performed SDH extension demonstrated a highly positive linear correlation between TGS and CCT (*r* = 0.962; *p* < 0.001). Looking at the different examination time points TGS and CCT demonstrated positive linear correlation at al time points (initial examination: *r* = 0.954, *p* < 0.001; first follow-up examination: *r* = 0.983, *p* < 0.001; second follow-up examination: *r* = 0.97, *p* < 0.001) (Figure 2). In 11 patients, acute evacuation of SDH due to SDH expansion was necessary. Sonographic measurement had a sensitivity of 90.9% and a specificity of 93.8% in predicting surgical intervention when sonographic extension of SDH exceeded above 13.25 mm (*p* < 0.001) (Figure 3).

## Discussion

Transcranial gray-scale sonography is the method of choice to diagnose and monitor extracerebral space enlargement in newborn and infants. In addition, distinction of benign subarachnoid space enlargement, hydrocephalus externus, bacterial meningitis, and SDH and hygroma is possible (4, 11). Differentiation of the subarachnoid and the subdural space is based on the depiction of vessels within the subarachnoid space and the depiction of an echogenic membrane. The echogenic membrane is the sonographic correlate of the dural border of the arachnoid, separating the subarachnoid from the subdural space (4, 11, 12). Also, some authors propagate intraoperative ultrasound in SDH evacuation to detect deep-seated isolated hematomas in need for fenestration during surgery (13) as well as complicating contralateral epidural or SDHs in decompressive surgery (14). Based on pediatric ultrasound experience, it has been demonstrated that visualization of SDH in adults is possible even in poor transtemporal bone window. Also, differentiation of acute and chronic SDH is possible and correlation of extension of SDH between CCT and TGS is high (8).

Treatment of SDH usually is surgical but conservative treatment is feasible in patients with small SDH extension and no sign of mass effect (1). Especially in chronic and partially acute on chronic SDH spontaneous resorption and consecutive regression have been described (15, 16). Criteria that qualify for conservative treatment are clinical as well as radiological (1). So far sequential CCT monitoring is the method of choice in monitoring SDH besides clinical evaluation (2). It has been shown that in some patients with traumatic head injuries, who have not undergone surgical intervention in the beginning, changes on serial CCT may precede clinical deterioration especially in extending SDH (17). In addition, changes on CCT with or without clinical deterioration are associated with a worse outcome thus indicating serial imaging (18). Despite this, CCT is a radiological method requiring patient transportation to the radiology suite and placing the patients at risk during transportation. Thus, there is a need for noninvasive bedside monitoring of conservatively treated SDH.

With the presented study we were able to show that correlation of TGS measured extension of SDH with CCT is high during the time course of SDH monitoring. In case of SDH expansion on CCT TGS detected expansion of SDH as well. These data are in accordance with data from pediatric ultrasound (11, 19, 20). In addition, these findings are in accordance with data from studies comparing the value of transcranial monitoring of intraparenchymal hematoma with CCT. In several studies, monitoring of intracerebral hematoma with ultrasound was possible and size, volume, and localization of intracerebral hemorrhage measured by TGS are highly acurate compared with CCT (5–7, 21). It was even possible to differentiate the different phases of hematoma resorption *via* TGS (5, 7). Although we did not monitor for changes of echogenicity of SDH, we were able to demonstrate that it is possible to monitor extension of SDH with TGS since correlation of TGS und CCT measurement of SDH extent remained high during follow-up. In accordance with a reliable detection rate of intracerebral hematoma volume expansion in several studies (6, 7), we were also able to clearly identify extensive SDH and SDH enlargement in need for surgical evacuation either primarily or during follow-up with a high sensitivity and specificity. Therefore, even though CCT is necessary for the surgical intervention, monitoring SDH may be performed with TGS up to this point since TGS shows reliable detection of patients in need for surgical evacuation of SDH. By adding the sonographic measurement of midline-shift comparable to studies on mass effect due to intracerebral hematoma or malignant stroke (22, 23) may allow an even closer monitoring of SDH expansion (24).

Our study on sonographic monitoring has some limitations that need to be addressed. Results are limited by the relatively small number of patients and follow-up examinations thus revealing progression of hematoma in need for surgical evacuation in a limited number of patients only. This might have influenced the high correlation between sonographically derived need for surgery and the clinical and radiological based indication for evacuation of hematoma. In addition, we did not monitor for other signs of hematoma-associated mass effects other than SDH extension, e.g., edema formation with midline shift. Consecutively, smaller SDH in need for surgical intervention due to progressive edema formation would have been missed. Therefore, further studies on combining sonographic parameters such as midline shift and compression of the ventricular system with TGS monitoring of SDH extension are needed in a larger set of patients for a more precise detection of SDH in need for surgery. Transcranial sonography and consecutively sonographic monitoring of SDH is limited due to an insufficient transtemporal bone window. Also, SDH localized in the posterior fossa or highly fronto-parietal and SDH progression within this region may not be detected due to the anatomy of the transtemporal bone window. In our study, SDH could not be depicted in 8.5% patients only. Furthermore, TGS is an investigator-dependent method and reliability of results relies on the experience of the investigator. Finally, the presented study describes the role of TGS in monitoring SDH diagnosed either by CCT or MRI. Thus, TGS may only serve as a monitoring tool at patients’ bedside, while the possibility of primary detection of SDH with TGS as described in infants remains unknown.

To summarize our results, we were able to show that monitoring of SDH with TGS is possible. TGS reliably identifies patients that may be in need for surgical evacuation of SDH, even though we did not monitor for secondary change due to SDH, e.g., ventricular compression or midline shift. Thus, TGS is an intriguing monitoring method especially in the critically ill since TGS may be performed at the patients’ bedside. Measurement of SDH *via* TGS is impossible in patients without transtemporal bone window only and thus may be performed in a high percentage of patients with SDH reducing transportation of critically ill patients as well as radiation strain due to serial CCT scanning.

## Ethics Statement

This study was carried out in accordance with the recommendations of the ethics committee of Hamburg University and Freiburg University, Germany with informed consent in accordance with the Declaration of Helsinki of all subjects or their legal guardians.

## Author Contributions

W-DN has carried out the investigation (acquisition of clinical, radiological, and sonographical data) described in the study and has drafted the manuscript as well as the figures submitted and thus mainly has contributed to the work presented. MR has performed part of the data acquisition (sonographic examination) and has added substantial revision to the manuscript. CW contributed to the conception and design of the study and has contributed substantial revision to the manuscript.

## Conflict of Interest Statement

None of the authors received any financial compensation for their contribution to this study. W-DN has received travel expense funding of Fresenius respectively with no potential conflict of interest. MR has no relationships/conditions/circumstances that present potential conflict of interest. CW in the past has received payment for consultancy for Pierre Fabre with no potential conflict of interest.
